# The Role of Immune Checkpoint Inhibitors in Metastatic Pancreatic Cancer: Current State and Outlook

**DOI:** 10.3390/ph16101411

**Published:** 2023-10-04

**Authors:** Linh Chi Tran, Berna C. Özdemir, Martin D. Berger

**Affiliations:** Department of Medical Oncology, Inselspital, Bern University Hospital, University of Bern, 3010 Bern, Switzerland

**Keywords:** pancreatic cancer, metastatic, immune checkpoint inhibitors

## Abstract

Pancreatic ductal adenocarcinoma (PDAC) is one of the deadliest tumors, characterized by its aggressive tumor biology and poor prognosis. While immune checkpoint inhibitors (ICIs) play a major part in the treatment algorithm of various solid tumors, there is still no evidence of clinical benefit from ICI in patients with metastatic PDAC (mPDAC). This might be due to several reasons, such as the inherent low immunogenicity of pancreatic cancer, the dense stroma-rich tumor microenvironment that precludes an efficient migration of antitumoral effector T cells to the cancer cells, and the increased proportion of immunosuppressive immune cells, such as regulatory T cells (Tregs), cancer-associated fibroblasts (CAFs), and myeloid-derived suppressor cells (MDSCs), facilitating tumor growth and invasion. In this review, we provide an overview of the current state of ICIs in mPDAC, report on the biological rationale to implement ICIs into the treatment strategy of pancreatic cancer, and discuss preclinical studies and clinical trials in this field. Additionally, we shed light on the challenges of implementing ICIs into the treatment strategy of PDAC and discuss potential future directions.

## 1. Introduction

Pancreatic ductal adenocarcinoma (PDAC) is one of the deadliest tumors with a median life expectancy of less than 10% at 5 years [1]. Pancreatic resection, followed by adjuvant chemotherapy, is currently the only curative intended option to achieve long-term survival [2]. 

However, only 20% of the patients with newly diagnosed PDAC present with localized disease amenable to surgery [3]. The remaining 80% do not qualify for surgery due to either locally advanced or metastatic disease [4]. Palliative first-line treatment options mainly consist of combination chemotherapy regimens, such as FOLFIRINOX (5-FU, folinic acid, irinotecan, and oxaliplatin) or gemcitabine (Gem), with or without nab-paclitaxel (NabP) depending on comorbidities, age, and performance status [5,6,7,8].

Within the next decade, the incidence of PDAC will steadily rise, and PDAC is estimated to become the second leading cause of cancer-related death by 2030 [9]. However, therapeutic options are still scarce and mainly based on combination chemotherapy with limited sustained efficacy. 

Additionally, effective biomarkers enabling us to identify patients who derive the most benefit from a specific treatment are still lacking. Over the last two decades, promising results from several early phase clinical trials assessing innovative therapeutic approaches have not been confirmed in phase III trials [10,11,12,13].

Several basket trials, including pancreatic cancer cohorts, evaluated the efficacy of targeted treatment options for various promising genetic alterations, such as mutations and gene fusions or rearrangements [14,15,16]. Whether the promising response rates to targeted treatment will translate into prolonged progression-free (PFS) and overall survival (OS) in mPDAC patients is still to be shown.

While ICIs are well established in several malignancies [17,18], they have not yet found their way into the treatment algorithm of pancreatic cancer.

In this review, we focus on the rationale for introducing ICI-based therapy in mPDAC patients and discuss the current role of ICIs in the treatment landscape of pancreatic cancer. Furthermore, we provide an overview of a selection of promising ongoing trials evaluating the impact of ICIs in mPDAC and shed light on the evolving role of immune therapeutic approaches in the future.

## 2. Rationale for Immune Checkpoint Inhibition in mPDAC

Several trials have investigated ICIs (alone or in combination with other drugs), cancer vaccines, and chimeric antigen receptor (CAR) T cell therapy [19,20,21] in mPDAC, without obtaining any improvement in patient survival.

Even in the presence of biomarkers that predict the efficacy of ICIs in most cancer types, PDAC remains evasive to ICIs. Genomic profiling of over 3500 PDAC samples has reported that 0.5% of the tumors were microsatellite instability-high (MSI-H) and/or tumor mutational burden-high (TMB-H) (≥20 mutations/Mb) [22]. While the objective response rate (ORR) to pembrolizumab was 34.3% among patients with mismatch repair deficient (dMMR)/MSI-high (MSI-H) non-colorectal tumors, the subgroup of PDAC had an ORR of 18.2% [23]. In another trial, the ORR was 0% among the 24 PDAC patients [24], suggesting the presence of unique resistance mechanisms in PDAC.

One key feature that distinguishes PDAC from other solid malignancies is the abundant desmoplastic stroma. Different types of fibroblasts (e.g., pancreatic stellate cells) are induced by cancer cells to deposit extracellular matrix (ECM) proteins surrounding the tumor cells. This desmoplastic tissue often comprises the bulk of the tumor mass and has been suggested to act as a physical barrier, resulting in hypoxia, insufficient drug penetration, and immune cell infiltration [25]. It was therefore assumed for a long time that the stromal reaction was tumor-promoting. Yet, it is now well established that the tumor microenvironment (TME) is very complex and also has tumor-restraining functions [25].

A plethora of genetically engineered mouse models (GEMMs) of PDAC allow for investigation into different constituents of the TME, the role of different genes in cancer initiation and progression, and screening for therapeutic targets and resistance mechanisms in immunocompetent mice [26]. These models are based on the Cre/loxP-mediated activation of mutant KRAS (G12D/V) in combination with the inactivation of one or more tumor suppressor genes (e.g., p53, Tgfrb2, Smad4, Ink4a/Arf) driven by the Pdx-1 or P48 promoter expressed by exocrine pancreatic cells. Depending on the inactivated tumor suppressor gene, these models recapitulate different characteristics of human PDAC [26].

GEMMs have been particularly useful in determining the role of the TME in PDAC. The reduction of the stroma by depletion of aSMA-expressing myofibroblasts or genetic deletion of sonic hedgehog (Shh), a critical factor for the formation of desmoplastic stroma, resulted in more aggressive tumor histology and decreased survival [27,28]. Importantly, reduced stroma resulted in an immunosuppressive TME with the emergence of Tregs and MDSC [27].

There is, in fact, considerable plasticity and morphologic and functional heterogeneity in stromal content. Histology-guided regional multiomics analysis of human pancreatic TME revealed “subTMEs”, tissue regions with distinct biological behavior. SubTMEs rich in fibroblasts and immune “hot”, with a high rate of CD8^+^ T cells, were classified as “reactive”. Those with abundant ECM but fewer fibroblasts and immune cells were classified as “deserted”. Consequently, while a deserted TME supported cancer cell differentiation, reactive states appeared to promote cancer progression through the proliferation of de-differentiated cancer cells [29], highlighting the context-dependent, alternating role of the TME. Mass cytometry analysis of pancreatic tissue from GEMMs showed dynamic changes in the immune cell content at different stages of cancer development and confirmed that immunosuppressive cells are already present at the initial phases of PDAC development [30]. As early as during acinar-to-ductal metaplasia (ADM), an accumulation of Tregs was observed, while effector T cells (Teffs) were largely missing, indicating that impaired adaptive immunity facilitates PDAC initiation, while at the metastatic stage, the recruitment of MDSCs and Arginin1(Arg1)-positive M2-type macrophages and the resulting decrease in innate immunity are the main drivers of immunosuppression, given the lack of infiltration by T or B cells. These findings were confirmed by immunohistochemistry of human PDAC samples [30]. Treatment with an Arg1-inhibitor synergized with anti-PD-1 inhibition in a GEMM. These results suggest that the currently available ICIs stimulating adaptive immunity might be more successful at earlier stages of PDAC [30]. It has been shown that KRAS G12D mutations inhibit antitumor immunity by attracting MDSCs [31] and tumor-associated macrophages (TAMs) into the precancerous tissue through the secretion of TNF and remodeling of the ECM cancer formation [32]. However, targeting KRAS G12D/V mutations might help to evade the immunosuppressive TME and improve the efficacy of ICIs in PDAC.

However, it might be not straightforward to identify new treatment options given that the TME can have, at the same time, tumor-promoting and tumor-restraining roles, depending on the cell type. Therefore, targeting of stromal elements is a double-edged sword that can have detrimental effects. Given the complexities of human PDAC in comparison to GEMM, several promising preclinical approaches yielded disappointing results in clinical trials. One prominent example of a stroma-targeting approach is pegylated recombinant human hyaluronidase 20 (PEGPH20), which significantly decreased intratumoral interstitial fluid pressure and increased vessel diameter in mice, resulting in improved chemotherapy delivery and almost doubled animal survival when given together with Gem [33]. However, in a phase Ib/II trial, patients treated with modified FOLFIRINOX (mFOLFIRINOX) had longer survival compared to those treated with mFOLFIRINOX plus PEGPH20, possibly related to the higher rate of serious adverse events and fewer chemotherapy cycles in the combination arm, resulting in lower chemotherapy exposure [34]. Although 7% of the patients treated with the PEGPH20 combination achieved complete remission, a very rare event in PDAC [34], a phase III trial testing PEGPH20 in combination with Gem/NabP did not improve overall survival [11], leading to discontinuation of the PEGPH20 program by the sponsor.

The analysis of paired human PDAC specimens before and after ICI treatment provided some insights into the sensitivity and resistance mechanisms as well as the alterations in the TME induced by ICIs. It was reported that a higher infiltration of tumor-associated neutrophils (TANs) expressing IL-8RB/CXCR2 upon neoadjuvant treatment with the vaccine GVAX and nivolumab was associated with poorer survival [35]. Interestingly, IL-8 is a cytokine secreted by pancreatic cancer cells in response to KRAS activation, and it is the main ligand for the IL-8RB/CXCR2 receptor of TANs, suggesting that anti-IL-8 antibodies might synergize with anti-PD-1 by inhibiting the chemotaxis of TANs [36]. Clinical trials testing this hypothesis are ongoing for several solid malignancies.

A meta-analysis demonstrated that mPDAC patients whose tumors displayed a high number of tumor-infiltrating lymphocytes (TILs) had improved OS, especially when the TILs were located in the tumor center [37], suggesting that strategies aimed at increasing the infiltration of CD8^+^ T cells might improve the response to ICIs. Several strategies were used to selectively reprogram the immunosuppressive TME.

The depletion of TAMs by inhibition of the CSF1R induces upregulation of PD-L1 and CTLA-4 expression, and ICIs in combination with CSF1R show greater antitumor responses compared to CSF1R inhibitors alone in orthotopic PDAC mouse models [38]. The results from clinical trials on various cancer types, including PDAC, are awaited.

Another promising approach is CD40-directed antibodies aimed at decreasing the stroma content and inducing TAMs to express major histocompatibility complex (MHC) class II molecules and the costimulatory molecule CD86 (B7-2), facilitating antigen presentation. CD40 antibodies have shown encouraging results in mouse models and in a small cohort of PDAC patients [39] and are currently tested in clinical trials with chemotherapy and/or ICI.

**Figure 1:**
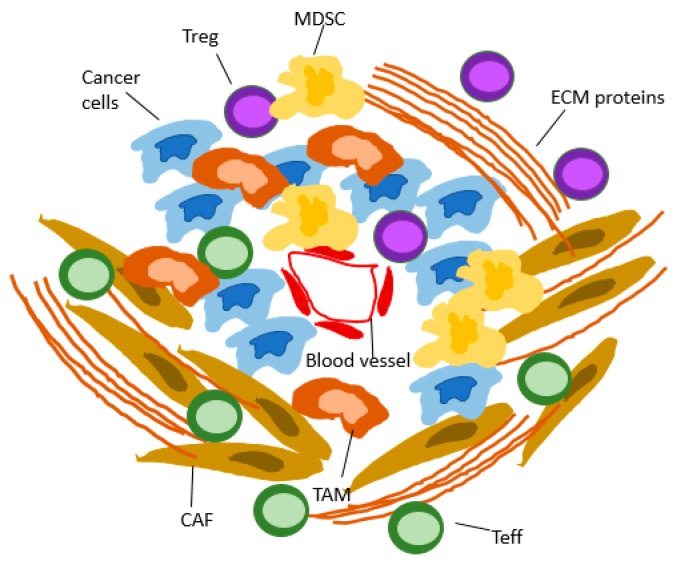
PDAC microenvironment with abundant cancer-associated fibroblasts (CAFs) and extracellular matrix (ECM). The stroma impedes the infiltration of lymphocytes (Teffs), while the TME recruits various immunosuppressive immune cells, such as myeloid-derived suppressor cells (MDSCs), tumor-associated macrophages (TAMs), and regulatory T cells (Tregs).

**Figure 2:**
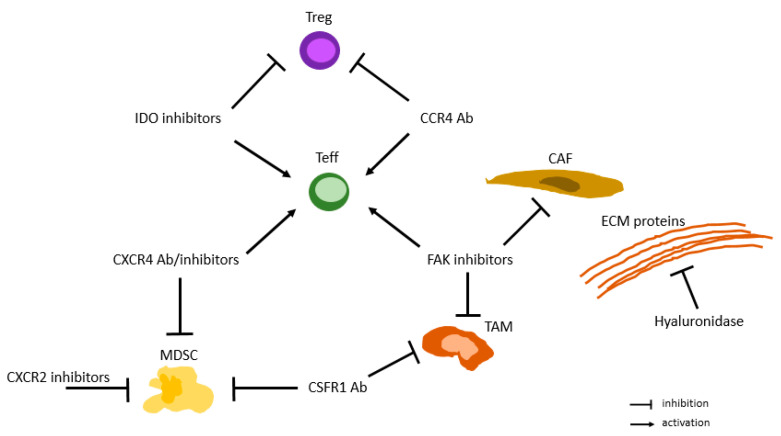
A selection of TME modulating agents and their inhibiting or activating effects on the TME components tested in mPDAC, either in preclinical models or clinical trials.

Taken together, these data show that there is considerable potential in TME-targeting approaches to optimize the efficacy of ICIs. The ideal time point for ICI treatment and the optimal combination of drugs need to be determined (Figure 1 and Figure 2).

## 3. Current Role of Immune Checkpoint Inhibitors in mPDAC

So far, ICIs do not play a role in the treatment of mPDAC patients. Several phase I-II trials exploring the efficacy of ICIs in combination with chemotherapy did not show any meaningful clinical benefit in advanced PDAC [40,41,42]. A selection of completed ICI-based phase II trials is outlined in Table 1.

The only encouraging phase II study was the PRINCE trial, demonstrating that combined treatment with nivolumab and Gem/NabP was correlated with a higher 1-year OS rate of 57.7% compared to a historical control (35%), *p* = 0.006. Interestingly, the combination of nivolumab and sotigalimab, an anti-CD40 antibody (Ab), plus Gem/NabP resulted in a 1-year OS of only 41.3% vs. 35% (historical control), *p* = 0.223. Similarly, the 1-year OS of sotigalimab plus chemotherapy was not significantly different from the historical control (48.1% vs. 35%, *p* = 0.062) [43].

At the ASCO Gastrointestinal Cancers Symposium 2022, Ueno et al. presented the overall response rates (ORRs) and OS data of a phase II trial investigating the efficacy of nivolumab in combination with mFOLFIRINOX in previously untreated mPDAC patients. The ORR, 1-year OS rate, and median OS (mOS) were 32.3%, 54.8%, and 13.4 months, respectively. Interestingly, but somewhat counterintuitively, was the longer mOS in CPS < 1 patients compared to those exhibiting a CPS ≥ 1 (13.5 vs. 8.2 months). However, the small cohort and the unplanned subgroup analysis do not allow for drawing any definite conclusions [44].

The Chinese phase II CISPD3 trial did not demonstrate a difference in the mOS between an ICI-based chemotherapy consisting of mFOLFIRINOX plus sintilimab and mFOLFIRINOX alone (10.9 vs. 10.8 months) [42].

The phase II KEYNOTE-158 study investigated the efficacy of pembrolizumab in pretreated MSI-H/dMMR metastatic noncolorectal cancer. Among 233 patients, 22 patients (9.4%) had pancreatic cancer. The ORR in patients with mPDAC was 18.2%, with a median PFS (mPFS) of 2.1 months and a mOS of 4.0 months [23].

In summary, there are still not sufficient data to justify the implementation of an ICI-based treatment for mPDAC patients in daily clinical practice.

### 3.1. Targeting PD-1/PD-L1 in mPDAC

One decade ago, a phase I study evaluated the efficacy and toxicity of an anti-PD-L1 Ab in several advanced solid tumors. While an objective response was observed in patients with melanoma, non-small cell lung cancer, and renal cell cancer, there was no antitumor activity in patients with pancreatic cancer [50]. This might be due to several reasons. First, the mutational load in pancreatic cancers is rather low compared to other solid tumors, leading to a decreased antigenicity with a low expression of tumor-specific proteins on the cancer cells [51]. Second, pancreatic cancer is characterized by an increased infiltration of immunosuppressive cells, such as MDSCs, TAMs, CAFs, and Tregs, resulting in an “immune desert-like” TME [52]. To increase immunogenicity, Weiss et al. designed a phase I trial to explore the efficacy of combined treatment with chemotherapy and the anti-PD-1 Ab pembrolizumab in patients with advanced solid tumors (*n* = 49) [53]. The addition of chemotherapy augmented tumor cell apoptosis, leading to an increased neoantigen presentation with subsequent stimulation of peritumoral CD8^+^ T cell attraction [53]. In this trial, 22.4% of the patients had pancreatic cancer (*n* = 11) and were treated with Gem/NabP and pembrolizumab. Only two patients had a partial response, resulting in an ORR of 18.2% [53]. Similarly, a recently conducted phase I study investigated the safety and efficacy of Gem/NabP and nivolumab in patients with locally advanced or mPDAC (*n* = 50). The ORR was 18% and almost identical to the rate of the previous study by Weiss et al. [54]. Moreover, the addition of nivolumab did not translate into a longer mPFS or mOS compared to the historical data of combined treatment with Gem/NabP [54]. Conversely, a small phase II pilot trial investigated the impact of an intensified treatment comprising nivolumab, Gem/NabP, cisplatin, and paricalcitol in patients with untreated mPDAC (*n* = 10) [55]. The rationale for adding the vitamin D analog paricalcitol was based on experimental studies demonstrating an inhibitory effect of vitamin D on MDSCs and Tregs that aimed at creating a more immunogenic TME. The ORR was 80% and the mPFS was 8.2 months, whereas the mOS has not yet been reached. Grade 3–4 toxicities were thrombocytopenia (100%) without bleeding events and colitis (20%) [55]. The KEYNOTE-028 was a phase Ib trial that included 475 patients with PD-L1 positive advanced solid tumors who underwent treatment with pembrolizumab. The primary endpoint was the ORR, and the secondary endpoints were safety and survival. Correlations among the T cell-inflamed gene expression profile, TMB and PD-L1 expression, and outcome were investigated to identify patients who benefitted the most from ICIs. Among 20 different cancer types, pancreatic cancer was the only tumor entity that did not show any response to pembrolizumab [24].

These results from several trials demonstrate that monotherapy with ICIs or ICIs combined with chemotherapy did not result in the improvement of clinical outcomes in mPDAC patients.

### 3.2. Targeting CTLA-4 in mPDAC

Preclinical studies revealed an increased amount of immunosuppressive cells in the TME of pancreatic cancer in immunocompetent mice [56]. Similarly, a predominant fraction of regulatory T cells was detected in the TME of human PDAC [57]. To overcome the immunosuppressive TME and to increase the anti-tumorigenic T cell response in PDAC, Royal et al. explored, more than a decade ago in a phase II trial, the tolerability and efficacy of ipilimumab monotherapy in patients with locally advanced or mPDAC. While the immune checkpoint therapy was safe, no clinical response was observed [49]. Based on preclinical studies suggesting a synergistic effect when combining anti-CTLA antibodies with granulocyte-macrophage colony-stimulating factor (GM-CSF) cell-based vaccines, a phase Ib study evaluated a combined treatment approach with ipilimumab with or without allogeneic pancreatic tumor cells transfected with a GM-CSF gene (GVAX) in patients with previously treated locally advanced or mPDAC [58]. While the combined therapy was well tolerated, no response to treatment was observed in either arm. The median OS was 3.6 months in the ipilimumab arm and 5.7 months in the ipilimumab/GVAX arm (HR: 0.51, 95% CI, 0.23–1.08, *p* = 0.072) [58]. Another phase I study demonstrated that the anti-CTLA-4 Ab tremelimumab combined with Gem was safe, with an ORR of 7.1% in previously untreated patients with mPDAC [59]. Kamath et al. designed a phase Ib trial to evaluate the safety and efficacy of a combined chemotherapy/ICI-based approach consisting of Gem and ipilimumab in locally advanced or mPDAC [40]. The primary endpoint of establishing the maximal tolerated dose (MTD) was met (Gem 1000 mg/m2 and ipilimumab 3 mg/kg). The combined treatment was well tolerated and safe, and the ORR, mPFS, and mOS were 18%, 2.8 (95% CI, 1.61–4.83), and 6.9 months (95% CI, 2.63–9.57), respectively [40]. A phase II randomized trial investigated the safety and efficacy of both a single and dual immune checkpoint treatment in patients with pretreated mPDAC [46]. The combination treatment, consisting of the PD-L1 monoclonal Ab durvalumab and the anti-CTLA-4 Ab tremelimumab, was well tolerated and yielded an ORR of 3.1%, while no patient showed a response with durvalumab monotherapy. The median PFS was equal in both treatment arms (1.5 months), and the mOS was 3.1 months in the dual treatment arm and 3.6 months in the durvalumab arm [46]. Another phase II study (*n* = 180) demonstrated that the addition of durvalumab and tremelimumab to Gem/NabP as a first-line treatment was not associated with an improved clinical outcome compared to Gem/NabP alone in patients with mPDAC (mOS: 9.8 vs. 8.8 months, HR: 0.94, 90% CI, 0.71–1.25, *p* = 0.72; mPFS: 5.5 vs. 5.4 months, HR: 0.98, 90% CI, 0.75–1.29, *p* = 0.91; and ORR: 30.3% vs. 23.0%, *p* = 0.28) [41].

To date, the implementation of anti-CTLA-4 Ab into the treatment algorithm of PDAC has not yet translated into a clinically meaningful improvement in outcome. This might be due to several reasons. Bengsch et al. demonstrated that CTLA-4 blockade resulted in increased tumoral CD4^+^ T cell infiltration in a mouse model. However, no increase in the CD8^+^ T cell count was observed within the TME to effectively exert an anti-tumor immune response [60]. Additionally, the immune escape mechanisms exerted by immunosuppressive cells, such as MDSCs, TAMs, and CAFs, may contribute to the lack of clinical activity of anti-CTLA-4 treatment. Therefore, novel treatment strategies that include targeted therapy focusing not only on tumor cells but also on the TME, combined with ICIs, need to be explored to further advance this field.

### 3.3. Targeted Treatment in Combination with ICI in mPDAC

#### PARP Inhibitors

Preclinical data suggest a synergistic anti-tumorigenic effect when combining PARP inhibitors with ICIs in pancreatic cancer that harbors mutations in the homologous recombination repair genes [61,62]. Just recently, Schram et al. demonstrated in a non-randomized tumor agnostic phase IIb basket study (*n* = 200) that patients with locally advanced or mPDAC (*n* = 16) displaying a germline or somatic mutation in BRCA1, BRCA2, or ATM and treated with the anti-PD-L1 Ab avelumab combined with the PARP inhibitor talazoparib yielded an ORR of 12.5% [63]. This result is comparable with that of the previous RUCAPANC phase II study investigating the efficacy of a PARP inhibitor monotherapy with rucaparib in patients with locally advanced and mPDAC (*n* = 19), ORR: 15.8% (*n* = 3) [64]. A randomized phase Ib/II trial evaluated the safety profile and outcome in patients with advanced PDAC who had stable disease after at least 4 months of platinum-based treatment and subsequently received niraparib plus nivolumab or niraparib plus ipilimumab [65]. The 6-month PFS, ORR, and mOS were 20.6%, 7.7%, and 13.2 months in the niraparib plus nivolumab arm (*n* = 44), and 59.6%, 15.4%, and 17.3 months in the niraparib plus ipilimumab group (*n* = 40) [65]. Interestingly, a post-hoc analysis revealed that the favorable outcome in the niraparib and ipilimumab cohort remained when focusing only on patients without any pathogenic BRCA1, BRCA2, or PALB2 variants (mPFS and mOS were 1.9 and 13.2 months in the niraparib plus nivolumab, and 7.6 and 17.3 months in the niraparib and ipilimumab arms, respectively) [65]. Due to these results, one may hypothesize that combined treatment with the PARP inhibitor niraparib and anti-CTLA-4 Ab ipilimumab can be used as a promising maintenance treatment in platin-sensitive advanced PDAC irrespective of whether BRCA1, BRCA2, or PALB2 variants are present or not. However, this early-phase trial was not designed to compare the efficacy of both treatment arms [65]. Therefore, a confirmatory phase III trial is needed to evaluate the impact of this maintenance treatment in our daily clinical practice. Currently, several phase II trials are ongoing to further evaluate the role of single or dual ICI in combination with PARP inhibition in patients with advanced PDAC that harbor mutations in genes encoding for homologous recombination repair [66,67,68].

### 3.4. ICIs in Combination with TME-Modulating Agents in mPDAC

#### 3.4.1. Immune Checkpoint Inhibition in Combination with Anti-CSF1R Ab

ICIs with anti-PD-1/PD-L1 or anti-CTLA-4 Ab have not yet led to an improvement in outcome in patients with mPDAC, irrespective of whether they were combined with backbone chemotherapy, such as Gem +/− NabP or FOLFIRINOX, or were administered as single or dual ICI therapy. Therefore, new avenues have to be explored to move forward in the treatment of PDAC. One potential strategy is to overcome resistance by targeting immunosuppressive cells, such as TAMs, to enhance the activation and recruitment of CD8^+^ cells [69]. Preliminary experimental work demonstrated that blocking the CSF1/CSF1R axis reprograms the TME by decreasing the number of TAMs and downregulating the genes involved in macrophage response in a syngeneic orthotopic mouse model [38]. Additionally, the remaining TAMs exhibited a decreased immunosuppressive gene expression profile, while anti-tumorigenic genes were upregulated [38]. Colony-stimulating factor 1 (CSF1) binds to its cognate receptor CSF1R and regulates the survival and migration of TAMs. Targeting CSF1R intends to deplete TAMs and render the TME into a less immunosuppressive milieu [38]. A phase II study tested combined treatment with an anti-CSF1R Ab and pembrolizumab in advanced solid tumors (*n* = 101), which are historically refractory to anti-PD-L1 therapy, and assessed the safety and activity of this novel approach. While the safety profile was acceptable, none of the 31 patients with mPDAC yielded an immune-related partial response (iPR) [70]. This result was confirmed by another phase I/II trial exploring the tolerability and the anti-tumoral activity of the same combination treatment strategy in patients with pretreated and refractory cancers (*n* = 57). Only one of the 27 mPDAC patients in the phase II part had a partial response (3.7%), while 7 patients presented with stable disease (25.9%) [71]. Another early-phase clinical trial demonstrated that combined treatment with the anti-PD-1 Ab nivolumab and the anti-CSF1R Ab cabiralizumab had a tolerable safety profile (*n* = 33) and achieved an ORR of 13% and a disease control rate (DCR) of 16% (*n* = 31) in patients with heavily pretreated mPDAC (*n* = 33) [72]. Based on these results, an ongoing trial is evaluating the clinical impact of cabiralizumab plus nivolumab with or without chemotherapy [73]. Given the preclinical data demonstrating that CSF1R inhibition induced not only PD-L1 but also CTLA-4 expression in CD8^+^ T cells, it might be promising to evaluate the efficacy of combined triple therapy consisting of anti-CTLA-4, anti-PD-L1, and anti-CSF1R Ab.

#### 3.4.2. ICI in Combination with FAK Inhibition

Focal adhesion kinases (FAKs) are tyrosine kinases comprising FAK1 and FAK2 and are involved in the regulation of cell adhesion and migration [74,75].

FAK expression is significantly higher in human PDAC compared to normal pancreatic tissue. Additionally, there is abundant literature demonstrating that an increased FAK1 expression is associated with poor outcomes in several malignancies. Experimental data indicate that FAK1 promotes stromal proliferation and suppresses CD8^+^ T cell activation in pancreatic cancer. FAK inhibition led to tumor regression, decreased stromal cell proliferation, and restoration of immune cell activity, translating into longer survival in a mouse model of pancreatic cancer [74].

These results warrant further exploration in clinical trials to assess the efficacy of combined ICI and anti-FAK treatment. Just recently, Wang-Gillam et al. designed a phase I dose-escalation and expansion study to explore the efficacy and safety of a FAK inhibitor (defactinib) in combination with pembrolizumab and Gem in previously treated patients with advanced pancreatic cancer. While the triplet therapy had a tolerable safety profile, only 1 out of 20 patients with refractory disease had a PR (5%). Similarly, one patient in the maintenance group (*n* = 10) obtained a PR (10%) [75]. Given the limited effect of this regimen, a different, more active backbone chemotherapy should be incorporated into this triplet treatment to increase the chance of an improved outcome.

#### 3.4.3. ICIs in Combination with a CD40 Agonist

CD40 is expressed in macrophages, B cells, dendritic cells, and several tumor cells and plays a major role in regulating the anti-tumoral T cell response [76]. More than a decade ago, Beatty et al. conducted a phase I clinical trial to test the efficacy of a CD40 agonist in combination with Gem in therapy-naïve patients with locally advanced or mPDAC (*n* = 21). Four patients achieved a PR (19%). Additionally, it was demonstrated that treatment with CD40 agonists resulted in T cell-independent tumor regression in immunocompetent mice. Interestingly, tumor shrinkage was mediated by CD40-activated TAMs [39,77].

Given these observations, a combined approach using anti-PD-1/PD-L1 inhibitors and a CD40 agonist to stimulate both T cells and TAMs might be effective in at least partially converting the immunosuppressive TME into a more immunogenic milieu and potentially overcoming resistance to anti-CTL4 or anti-PD-L1 therapy. Just recently, a phase I trial investigated the efficacy and tolerability of a first-line combination treatment consisting of a CD40 agonist (sotigalimab) and Gem/NabP with or without nivolumab in patients with mPDAC [78]. The subsequent phase II part, already mentioned above, randomized patients to treatment with nivolumab and chemotherapy (*n* = 34), sotigalimab and chemotherapy (*n* = 36), or nivolumab, sotigalimab, and chemotherapy (*n* = 35) [43]. Contrary to expectations, patients in the combined arm receiving the anti-PD-1 Ab, CD40 agonist, and chemotherapy did not have longer survival compared to those who were treated with nivolumab and chemotherapy or sotigalimab and chemotherapy [43].

Interestingly, the triple combination resulted in an increase in circulating B regulatory cells (CCR7+ CD11b+ CD27-), translating into a shorter OS [43]. Additionally, preclinical data on glioma suggest that CD40 agonists impede the efficacy of ICIs by upregulating B regulatory cells (Bregs) [79]. Therefore, one may hypothesize that Bregs exert an immunosuppressive effect after dual treatment with CD40 agonists and ICIs. However, further studies have to be conducted to elucidate the role of Bregs in promoting anti-tumorigenic effects in mPDAC.

#### 3.4.4. ICIs in Combination with an IDO Inhibitor

Indeolamin 2,3-dioxygenase 1 (IDO1) is a rate-limiting enzyme that degrades tryptophan into kynurenine. It suppresses CD8^+^ T cell function, stimulates Treg activation, and is upregulated in various malignancies [80]. Furthermore, increased IDO1 expression is associated with shorter OS in several solid tumors, such as colorectal cancer and pancreatic cancers [80,81]. Due to its immunosuppressive effects, targeting IDO1 may be promising to enhance antitumor immune response and increase the immunostimulatory effects of ICIs. A phase Ib study assessed the safety and tolerability of navoximod, a small-molecule IDO1 inhibitor, combined with the anti-PD-L1 ICI atezolizumab in patients with pretreated advanced cancers [82]. While the treatment was well tolerated, the efficacy was rather low, with an ORR of 9% in the dose-escalation part, and 11% in the expansion cohort. Only one patient with PDAC was enrolled in this trial (<1%). However, this patient achieved a partial remission and remained more than 650 days under treatment [82]. Another phase I/II study evaluated the safety and effect of combining the anti-PD-L1 Ab durvalumab with the IDO1 inhibitor epacadostat in patients with metastatic solid tumors, including 15 patients with mPDAC who were only enrolled in the phase I part of the study [83]. While the safety profile of the combination treatment was acceptable, the phase II part of the study was prematurely closed [83] after the phase III KEYNOTE-252 study of pembrolizumab and epacadostat vs. pembrolizumab plus placebo did not show any difference in PFS or OS in patients with metastatic melanoma [84].

#### 3.4.5. ICIs in Combination with an Anti-CCR4 Ab

The chemokine receptor 4 (CCR4) is expressed on Th2 cells and Tregs. Binding of the ligands CCL17 and CCL22 to their cognate receptor CCR4 enhances the suppressive activity of Tregs [85]. Several studies have indicated that an increased infiltration of Tregs in the TME was associated with poor clinical outcomes in several malignancies [86]. Tregs play a major role in maintaining an immunosuppressive TME by suppressing anti-tumorigenic immune responses. Moreover, Tregs contribute to the resistance mechanisms of anti-PD-1/PD-L1 therapy. Therefore, adding a Treg-depleting anti-CCR4 Ab to an anti-PD-1/PD-L1 treatment might be promising to overcome the immunosuppressive barrier and to improve responsiveness to ICI therapy in patients with PDAC. A phase I trial tested the tolerability and efficacy of a combined treatment strategy comprising nivolumab and the anti-CCR4 Ab mogamulizumab in patients with previously treated progressive solid tumors (*n* = 96) [87]. A total of 15 patients with pancreatic cancer were included in the study. One patient achieved a PR, whereas an unconfirmed response was observed in two patients. An exploratory immune cell subset analysis revealed a post-treatment increase in the proportion of CD8^+^ cells and a decreased amount of Tregs [87]. Another phase I study explored the anti-CCR4 Ab mogamulizumab with either durvalumab or tremelimumab in patients with advanced solid malignancies [88]. Only patients with PDAC were enrolled in the dose expansion cohort (*n* = 24). No patient in either treatment arm achieved a response [88].

#### 3.4.6. Targeting the CXCL12/CXCR4 Axis in Combination with ICIs

The CXCL12/CXCR4 axis is well known for its role in hematopoietic stem cell homing. Plerixafor, a CXCR4 antagonist, is routinely used to mobilize hematopoietic stem cells before autologous stem cell transplantation in patients with multiple myeloma and non-Hodgkin lymphoma [89,90]. Just recently, experimental studies have further explored the impact of the CXCL12/CXCR4 pathway on the immunosurveillance of various solid tumors. Fleig et al. demonstrated that, despite the presence of CD8^+^ T cells, dual ICI with anti-CTLA-4 Ab and PD-L1 did not exert a meaningful tumor response in a murine PDAC model [91]. However, depletion of the fibroblast activation protein (FAP) produced by CAFs rendered tumors more susceptible to ICIs [91]. This observation suggests that the immunosuppressive activity of CAFs contributes to the failure of ICIs in murine PDAC, and thus, plays a major role in tumor immune evasion. Treatment with a CXCR4 antagonist promoted CD8^+^ T cell recruitment to the tumor. By adding an anti-PD-L1 agent to the CXCR4 blockade, a significant decrease in cancer cells was observed, suggesting a synergistic effect when combining ICIs with CXCR4 or CXCL12 antibodies [91].

Moreover, Seo et al. demonstrated that dual blockade of PD-1 and CXCR4 not only triggered migration of CD8^+^ T cells from the tumor stroma to the close proximity of tumor cells but also enhanced its ability to kill tumor cells in live slice cultures of human PDAC [92]. According to their findings, they postulate, contrary to the widespread opinion, that there is no lack of activated T effector cells in PDAC but rather a spatial redistribution of already clonally expanded and activated anti-tumoral T cells within the stromal area, away from the proximity of tumor cells that impede a direct and efficient CD8^+^ T cell-tumor interaction. Through targeting the CXCL12/CXCR4 axis in combination with PD-1 blockade, an efficient antitumor immune response can be achieved [92].

O’Hara et al. conducted a small phase I dose-escalating study investigating the maximum tolerated dose (MTD) and tolerability of a CXCR4 antagonist in combination with the anti-PD-L1 Ab durvalumab in treatment-refractory advanced cancers (*n* = 9) [93]. Eight of nine patients had pancreatic cancer. No dose-limiting toxicities occurred, and the study treatment was well tolerated. However, no confirmed complete or partial response was observed among this heavily pretreated patient population [93].

A phase IIa trial explored the safety profile and the efficacy of the CXCR4 antagonist motixafortide combined with ICI (pembrolizumab) in patients with pretreated mPDAC [94]. The study treatment was considered to be safe, and the ORR and DCR were 3.4% and 34.5%, respectively. The mOS was 3.3 months in the overall population, whereas mPDAC patients receiving the study treatment in the second-line setting achieved a mOS of 7.5 months [94]. Blocking the CXCR4 axis induced a decrease in circulating Tregs and an increase in peripheral CD4^+^ T cells. No change in circulating natural killer (NK) cells was observed [94]. As expected, dual CXCR4 and PD-1 blockade was associated with an increased density of intratumoral CD8^+^ cells and a decreased amount of MDSCs [94]. Given the results of the dual CXCR4 and ICI blockade, an expansion cohort was activated to investigate a triple treatment with the anti-CXCR4 agent and PD-1 Ab combined with chemotherapy. The expansion cohort included mPDAC patients who developed disease progression after a Gem-based first-line treatment [94]. The second-line backbone chemotherapy consisted of nanoliposomal irinotecan (nal-IRI), 5-FU, and leucovorin. The ORR, DCR, and median DOR were 32%, 77%, and 7.8 months, respectively [94]. Due to these promising results, this experimental treatment approach should be further evaluated in a randomized phase III trial.

The OPERA phase I/II trial examined the safety and efficacy of the anti-CXCL12 inhibitor olaptesed pegol (NOX-A12), an L-RNA aptamer, as monotherapy, followed by a combination with pembrolizumab in a pretreated patient population with metastatic colorectal cancer (mCRC) or mPDAC [95]. Nine heavily pretreated patients with microsatellite stable (MSS) PDAC and a median of three lines of prior therapy were enrolled in this trial. While the study treatment was well tolerated, the ORR was 0%. The DCR in the mPDAC group was 22%. Remarkably, the duration of study treatment was quite long compared to the patients’ previous lines of therapy [95].

#### 3.4.7. Targeting CXCR2 in Combination with ICIs

CXCR2 is a G protein-coupled receptor that modulates the infiltration of MDSCs in the TME of PDAC [96]. Preliminary data indicate that high CXCR2 gene expression was correlated with poor outcomes in a small cohort of patients with resected PDAC. Steele et al. demonstrated that inhibition of CXCR2 diminished metastatic spread and improved survival in KPC mice. Interestingly, targeting CXCR2 resulted in increased sensitivity to ICIs, leading to prolonged survival in mice that received anti-CXCR2 and ICIs compared to mice treated with ICIs alone [96]. In line with this, Ullman et al. demonstrated that mice treated with FOLFIRINOX combined with an anti-CXCR1/CXCR2 inhibitor and an ICI had significantly longer survival than those who received FOLFIRINOX and anti-PD-L1 alone [97].

#### 3.4.8. ICIs Combined with Anti-Stromal Treatment

Pancreatic cancer is not only characterized by its immunosuppressive but also its dense and stroma-rich environment that prevents intratumoral accumulation of CD8^+^ T cells and aneffective delivery of chemotherapeutics to the tumor cells. One of the constituents of the extracellular matrix is hyaluronan [98]. Preliminary data demonstrated that an increased amount of hyaluronan in the stroma was associated with poor clinical outcome in patients with PDAC [99]. A randomized phase Ib/II study evaluated the safety and efficacy of combined treatment with pegylated recombinant human hyaluronidase (PEGPH20) and mFOLFIRINOX (experimental arm) vs. mFOLFIRINOX alone as first-line treatment in patients with mPDAC [34]. Noteworthily, there was an increased rate of grade 3/4 thromboembolic events in the experimental vs. control arms (10% vs. 2%). Additionally, the trial was prematurely closed, as the experimental arm yielded a shorter OS and PFS compared to mFOLFIRINOX alone (7.7 vs. 14.4 months, *p* < 0.01 and 4.3 vs. 6.2 months, *p* = 0.01, respectively) [34]. Interestingly, a randomized phase II study compared pegvorhyaluronidase alfa in combination with Gem/NabP vs. Gem/NabP in a first-line setting [10]. Metastatic PDAC patients with high expression of hyaluronan treated in the experimental PEGPH20 arm had a longer mPFS than those receiving Gem/NabP (9.2 vs. 5.2 months, HR: 0.51, 95% CI, 0.26–1.00, *p* = 0.048) [10].

Due to these promising results, a confirmatory phase III trial was initiated to compare Gem/NabP +/− pegvorhyaluronidase in first-line, hyaluronan-high mPDAC patients [11]. Unfortunately, no difference in mOS was observed between both treatment arms (11.2 vs. 11.5 months) [11]. Given the encouraging results from experimental studies showing that PEGPH20 increased the activity of ICIs in a murine model of cancer [100], Zhen et al. conducted a phase II trial evaluating the safety profile and efficacy of pembrolizumab combined with PEGPH20 in patients with treatment-refractory mPDAC and high expression of hyaluronan [101]. The trial accrual was prematurely stopped due to the negative results of the HALO-301 study [101]. While the treatment with pembrolizumab and PEGPH20 was not associated with an improved mPFS (1.5 months) compared to historical results, the mOS of 7.2 months was promising [101].

#### 3.4.9. ICIs in Combination with Anti-TGFbeta

Transforming growth factor-beta (TGFbeta) is a cytokine that can act as either a tumor promotor or suppressor depending on the tumor stage. While TGFbeta inhibits tumor growth in early-stage disease, it promotes tumor proliferation, migration, and invasion in late-stage cancers [102]. However, in addition to its dual effects on epithelial cells, TGFbeta also exerts stimulatory effects on stromal cells, leading to increased fibrosis, while simultaneously inhibiting immune stimulation, which further promotes tumor development [102]. Targeting both PD-L1 and TGFbeta might be an effective approach to improving anti-tumorigenic immune response in advanced and mPDAC, as both pathways exert non-redundant immunosuppressive effects. Preclinical studies demonstrated that a dual blockade of PD-L1 and TGFbeta resulted in T cell-mediated shrinkage of PDAC in a mouse model [103,104]. Subsequently, a phase I study examined the safety and efficacy of a bifunctional fusion protein containing an anti-PD-L1 Ab conjugated to the extracellular domain of the TGFbeta receptor in previously treated advanced cancers (*n* = 19) [105]. 26.3% of the enrolled patients (*n* = 5) had pancreatic cancers. Among these, one patient with MSI-H cancer achieved a PR [105]. Similarly, a phase I trial examined the clinical activity of a bifunctional anti-PD-L1/TGF-βRII agent in treatment-refractory advanced or metastatic solid tumors (*n* = 171) [106]. In patients with PDAC (*n* = 10), no clinical response was observed [106]. Another phase I trial assessed the efficacy of the TGFbeta receptor I kinase inhibitor galunisertib combined with durvalumab in patients with refractory mPDAC [107]. Similar to the previous trial, only one of 32 patients had a PR (ORR: 3.1%). The mPFS and mOS were 1.9 and 5.7 months, respectively. Again, while the safety profile was tolerable, the clinical outcomes were limited [107].

#### 3.4.10. ICIs in Combination with Bruton Tyrosine Kinase Inhibitor

Bruton tyrosine kinase (BTK) is a B cell and macrophage kinase, but it is also expressed in mast cells and platelets [108]. Ibrutinib, an inhibitor of BTK, is approved for the treatment of chronic lymphatic leukemia and mantle cell lymphoma and is also known to inhibit mast cell degranulation [109,110,111]. Strouch et al. demonstrated that increased mast cell infiltration in patients with PDAC was associated with a shorter OS [111]. The same group also showed that the presence of mast cells is a prerequisite for angiogenesis and tumor growth in a murine pancreatic cancer model [112]. Masso-Valles demonstrated that ibrutinib did not prevent the recruitment of mast cells to the tumor stroma but inhibited their degranulation in a pancreatic cancer mouse model [113]. Additionally, treatment with ibrutinib resulted in a significant decrease in fibrosis. Further experiments showed that mast cells were capable of inducing collagen accumulation in the stromal network of a murine pancreatic cancer model, suggesting that the anti-tumoral stroma effect of the BTK inhibitor ibrutinib relies on the presence of mast cells [113]. To overcome the obstacle of the dense fibrotic TMEs of pancreatic cancers, which inhibit an effective penetration of chemotherapeutics, the addition of the anti-fibrotic agent ibrutinib might be promising to improve the outcome of pancreatic cancer. Masso-Valles demonstrated that mice treated with ibrutinib had longer survival than the control group. Moreover, the addition of ibrutinib to Gem resulted in improved survival compared to treatment with Gem alone [113]. Gunderson et al. demonstrated that BTK and PI3K activation in B cells, MDSCs, and macrophages played a major role in the downregulation of CD8^+^ T cells, not only in mouse models of PDAC but also in human PDAC [108]. Additionally, inhibition of the BTK and PI3K signaling pathways with or without Gem resulted in an increase in T cells and in decelerated tumor growth. Depletion of T cells abrogated the beneficial effect of the combined treatment. These observations support further investigation into an ICI-based treatment combined with BTK [108]. A phase Ib/II basket study demonstrated that the combination of ibrutinib with durvalumab displayed a tolerable safety profile in pretreated mPDAC, breast cancer, and non-small-cell lung cancer patients, while the efficacy of this combined treatment was limited [114]. A randomized phase II study demonstrated that treatment with the BTK inhibitor acalabrutinib in combination with the anti-PD-1 Ab pembrolizumab in pretreated metastatic or locally advanced unresectable PDAC was tolerable and safe (15.8% grade 3–4 treatment-related adverse events with the dual therapy vs. 14.3% with acalabrutinib alone) [115]. However, the ORR and DCR were only modest in both treatment arms (7.9% and 21.1% with the combined treatment and 0% and 14.3% with the BTK inhibitor alone, respectively). Additionally, no difference in mPFS or mOS was seen between the combined BTK inhibitor plus pembrolizumab arm and the BTK inhibitor monotherapy (1.4 vs. 1.4 months and 3.8 vs. 3.6 months, respectively) [115]. In the phase III RESOLVE trial, the addition of ibrutinib to Gem/NabP was not associated with a difference in the mOS compared to placebo plus Gem/NabP (9.7 vs. 10.8 months, *p* = 0.3225) [116]. Moreover, patients treated with Gem/NabP plus placebo had a longer mPFS compared to those receiving Gem/NabP and ibrutinib (6.0 vs. 5.3 months, *p* < 0.0001) [116]. In summary, we can conclude that, despite promising experimental data, several clinical trials have failed to demonstrate a survival benefit when adding ibrutinib to ICIs or chemotherapy. Therefore, new ways have to be explored to determine how to best implement BTK inhibitors in the treatment algorithm of mPDAC.

### 3.5. Combination of Immunomodulating Agents

#### 3.5.1. Anti-PD-1 Ab in Combination with OX40 Agonists

Experimental work demonstrated that combined treatment with an anti-PD-1 Ab and an anti-OX40 agonist resulted in longer survival compared to monotherapy with either anti-PD-1 Ab or anti-OX40 in a pancreatic cancer mouse model [117]. Furthermore, it has been shown that the antitumor immune response elicited by the ICI combined with an OX40 agonist was completely dependent on the presence of CD4^+^ T cells, partially CD8^+^ T cell-dependent, and independent of the presence of NK cells. The combined treatment approach resulted in an increase in the total amount of CD4^+^ T cells and in an expansion of memory CD8^+^ and CD4^+^ T cells, while the proportion of Tregs declined [117].

These observations confirm again that a single ICI is not enough to induce an effective immune response in pancreatic cancer. Moreover, this experimental study indicates that at least a synergistic treatment consisting of an anti-OX40 agonist and a PD-1 antagonist is required to circumvent the obstacles of an immunosuppressed TME in PDAC.

However, clinical trials need to be performed to definitely prove the efficacy of this combined treatment approach in patients with mPDAC.

#### 3.5.2. Chemotherapy in Combination with an Anti-LAG-3 Ab

The lymphocyte-activation gene 3 (LAG-3) is mainly expressed on CD8^+^ and CD4^+^ T cells and plays a major role in downregulating T cell activation [17]. A phase I trial investigated the MTD of IMP321 and Gem [118]. IMP321 is a dimeric recombinant fusion protein of LAG-3 that binds to the MHC II of antigen-presenting cells (APC), resulting in CD8^+^ activation. The combination treatment did not cause any severe adverse events. However, no incremental efficacy was observed when IMP321 was added to Gem, most likely due to the suboptimal dosing of the investigated product [118]. Further clinical trials are warranted to evaluate the safety and efficacy of a combined anti-PD-1/PD-L1 and anti-LAG-3 treatment approach +/− chemotherapy [118].

#### 3.5.3. Targeting TIGIT

T cell immunoglobulin and ITIM domain (TIGIT) is a co-inhibitory receptor expressed on various immune cells, such as activated T cells, NK cells, memory T cells, and Tregs. The ligands of TIGIT are CD155 and CD112, which are expressed on APCs but also on tumor cells [119]. Preclinical models demonstrated that the CD155/TIGIT axis reinforced immune cell evasion in pancreatic cancer [120]. Blocking of TIGIT and PD-1 in combination with a CD40 agonist has been shown to effectively stimulate an anti-tumoral T cell response in a pancreatic mouse model [119].

Despite accumulating promising data from animal studies, there are currently no clinical trials exploring the impact of anti-TIGIT Ab on mPDAC. However, targeting this immune-inhibitory axis might be a promising approach to optimize treatment against mPDAC.

#### 3.5.4. Targeting VISTA

The V-domain immunoglobulin suppressor of T cell activation (VISTA) is an inhibitory immune checkpoint molecule that is mainly expressed in macrophages [121]. Blando et al. demonstrated that VISTA expression is increased on CD68+ macrophages in pancreatic cancer [122]. Additionally, they observed that the density of immune cells expressing VISTA in the TME is significantly higher in pancreatic cancer compared to melanoma. Interestingly, activation of the VISTA pathway resulted in a more pronounced decrease in CD8^+^ T cell-mediated immune response than stimulation of the PD-L1 axis. Therefore, PD-L1 blockade may not restore antitumor immunity in pancreatic cancer, as VISTA still exerts its inhibitory effects by inducing downregulation of cytokine production in TILs [122]. Thus, the implementation of anti-VISTA in addition to anti-PD-L1 blockage Ab might be helpful for optimizing the immune response in patients with pancreatic cancer.

#### 3.5.5. ICIs in Combination with MET Kinase Inhibitors

A recently conducted phase I trial investigated the efficacy of an anti-PD-L1 Ab with and without other targeted agents in patients with previously treated cancers (*n* = 61) [123]. One of these drugs was merestinib, a MET kinase inhibitor that blocks several oncogenic key drivers, such as FLT3, AXL, and MKNK1, which mediate the immunosuppressive effects of MDSCs, and thus, contribute to the immunologically “cold” TME in pancreatic cancers [123]. Combining ICIs with a MET kinase inhibitor may result in a synergistic anti-tumorigenic response by increasing the T cell response and decreasing the immunosuppressive effects of MDSCs in the pancreatic TME.

Patients with pancreatic cancer were treated in the merestinib combination arm (*n* = 17). While 1 out of 5 patients in the dose-escalation cohort achieved a PR (20%), none of the 12 patients allocated to the expansion cohort had a PR (ORR: 0%) [123].

#### 3.5.6. ICIs Combined with a STING Agonist

The stimulator of interferon genes (STING) pathway plays a crucial role in generating type I interferons that activate anti-tumoral T cells. Ager et al. demonstrated that a STING agonist further enhanced the efficacy of dual ICI in a murine model of PDAC [124]. Moreover, it was observed that the STING agonist inhibited the expansion of murine MDSCs through downregulation of c-myc. Combined treatment with the STING agonist and ICI resulted in complete tumor regression, translating into a longer OS compared to mice treated with the ICI or STING agonist alone [124]. Additionally, treatment with a STING agonist in combination with an ICI not only increased the CD8^+^ effector T cell-mediated immune response but also induced reprogramming of MDSCs and TAMs into more proinflammatory phenotypes [124]. This remodeling of the TME and the stromal compartment may contribute to overcoming the hurdles of an immunosuppressive “cold” PDAC and facilitating the efficacy of ICIs.

#### 3.5.7. Immunomodulating Triplet Treatment

Since single and dual ICI did not translate into improved clinical outcomes in several trials, new ways have to be explored to optimize the treatment armamentarium against pancreatic cancer. Considering the stroma-rich TME with abundant immunosuppressive cells, such as Tregs, MDSCs, TAMs, and CAFs, a combined therapeutic approach comprising ICIs and agents simultaneously targeting immunosuppressive cells might be promising to increase the clinical response and to prolong survival in patients with PDAC. Just recently, Gulhati et al. performed extensive immune and single-cell RNA sequencing profiling in murine and human pancreatic cancers to better delineate and decipher the tumor immune microenvironment (TIME) and its dynamic changes under treatment with various ICIs [125]. They observed increased expressions of two immune checkpoints, LAG-3 and 41BB, on exhausted T cells. A combined treatment with a 41BB agonist and a LAG-3 antagonist decreased tumor growth and prolonged survival compared to monotherapy with either the antibody alone or another ICI. At the baseline, the TIME comprised abundant MDSCs that expressed CXCR2. The blockade of CXCR2 impaired MDSC migration and activation, thereby leading to tumor growth inhibition [125]. By adding a CXCR2 inhibitor to the dual ICI, impressive tumor control and longer OS were observed in the majority of preclinical models. However, whether this triple regimen is safe and facilitates the transition of a non-immunogenic tumor into a “hot” tumor that can be rendered vulnerable needs to be further explored in a clinical trial [125].

### 3.6. Other Immunomodulating Therapeutic Approaches

#### 3.6.1. Immune Checkpoint Inhibition in Combination with Oncolytic Viruses

To date, several attempts have failed to render the immunosuppressive TME of pancreatic cancers more immunogenic. There is mounting evidence that the intratumoral administration of oncolytic viruses may increase the neoantigen load through tumor cell destruction. The so-called immunogenic cell death is characterized by an enhanced tumoral neoepitope presentation that attracts activated CD8^+^ cells, and thus, elicits an anti-tumorigenic immune response. A phase Ib trial investigated the efficacy and safety of an intravenously administered oncolytic virus (pelareorep) combined with pembrolizumab and chemotherapy in pretreated mPDAC patients (*n* = 11) [126]. One patient achieved a clinical response (ORR: 9%), and the mPFS and mOS were 2.0 and 3.1 months, respectively. It was demonstrated that pelareorep replicated in the tumor tissue and stimulated T cell migration to the TME [126]. The ongoing phase I/II GOBLET study is investigating the safety profile and efficacy of pelareorep in combination with atezolizumab with and without chemotherapy in several advanced or metastatic gastrointestinal malignancies, including pancreatic cancer [127].

#### 3.6.2. Immune Checkpoint Inhibition in Combination with mRNA-Based Vaccines

Various combined treatment approaches with ICI and targeted treatment or chemotherapy have failed to show any meaningful clinical benefit in patients with mPDAC. A phase I clinical trial assessed the efficacy and safety of an adjuvant treatment comprising a mRNA neoantigen vaccine (autogene cevumeran) combined with the anti-PD-L1 Ab atezolizumab and mFOLFIRINOX in patients with resected PDAC [128]. The combination treatment was well tolerated and safe. Moreover, autogene cevumeran induced a significant T cell response in 50% of the patients, translating into longer recurrence-free survival compared to those patients who did not develop adequate T cell expansion [128].

Whether these findings of increased T cell activation upon administration of mRNA cancer vaccines in an adjuvant setting are transferable to patients with mPDAC remains to be elucidated.

The implementation of mRNA-based vaccines combined with ICIs into the treatment algorithm of PDAC might open a new era of personalized therapy in patients suffering from mPDAC. However, randomized clinical trials are eagerly awaited to confirm these preliminary, promising results.

### 3.7. Local Treatment Combined with Immune Checkpoint Inhibitors

#### 3.7.1. Radiotherapy in Combination with ICIs

Given the low immunogenicity of pancreatic cancer, several clinical trials using single or dual ICI with and without chemotherapy or targeted therapy have failed to demonstrate any clinically meaningful benefit. Therefore, new treatment approaches are eagerly awaited to improve outcomes in patients with mPDAC. Radiotherapy induced tumor cell necrosis, leading to increased antigen presentation, and thereby, promoting immunomodulatory effects [129]. In addition to its direct and locally limited cytotoxic effect, radiotherapy also exerted a systemic anti-tumoral response that may have had an additional effect on distant sites (abscopal effect) [130]. However, radiotherapy also stimulated the migration of immunosuppressive cells, such as Tregs and MDSCs, to irradiated tumor sites, which mitigated immunostimulatory effects [131]. Azad et al. demonstrated that high-intensity radiotherapy resulted in enhanced intratumoral CD8^+^ T cell accumulation in a murine pancreatic cancer model [132]. By adding an anti-PD-L1 Ab, the proportion of CD8^+^ T cells in the tumor was further increased, while the amount of MDSCs was reduced [132]. Findings in mouse models demonstrated that PD-L1 expression in tumor and dendritic cells was increased after radiation compared to the PD-L1 levels of the identical cell types in control tumor tissue that had not been previously irradiated. However, the amount of PD-L1 expression on MDSC did not alter after radiation [133]. Furthermore, the impact on tumor shrinkage was higher in mice that underwent a combination treatment with radiation and PD-L1 inhibition compared to those receiving either radiation or anti-PD-L1 Ab alone [133]. Additionally, combined radio-/ICI therapy resulted in downsizing of a contralateral implanted secondary tumor, whereas no anti-tumoral effect was observed at a distant site with either treatment alone [133]. These findings suggest that combined radio-/ICI therapy not only exerts a local effect but also induces an abscopal effect at distant tumor sites. Moreover, it was demonstrated that the presence of CD8^+^ T cells is mandatory for the effectiveness of radio-/ICI therapy and that the combined treatment stimulates CD8^+^ T cell activation, which leads to a decreased accumulation of MDSCs [133]. These observations confirm the importance of combining different treatment modalities to modify the immune texture in the TME to further optimize cancer treatment. Combined treatment with radiation, Gem, and anti-PD-L1 Ab resulted in a significant delay of tumor growth compared to any single or double treatment combination in a syngeneic pancreatic cancer mouse model. Furthermore, radiotherapy plus anti-PD-L1 Ab increased the amount of CD8^+^ T cells and the CD8^+^/Treg ratio and not only exerted a local but also an antimetastatic, so-called abscopal effect [133]. This knowledge derived from animal studies led to the design of clinical trials in patients with mPDAC. Chen et al. designed the phase II randomized CheckPAC trial to assess the clinical benefit of nivolumab +/− ipilimumab combined with stereotactic body radiotherapy (SBRT) as further-line treatment in patients with mPDAC (*n* = 84) [134]. The clinical benefit rate, defined as the proportion of patients who achieved a complete response (CR), PR, or stable disease, was 17.1% in the SBRT/nivolumab arm and 37.2% in the SBRT/nivolumab/ipilimumab group. One patient in the SBRT/nivolumab arm (2.4%) and six patients enrolled in the SBRT/nivolumab/ipilimumab arm (14.0%) had a PR. The mOS and mPFS were equal in both arms (3.8 vs. 3.8 months and 1.7 vs. 1.6 months, respectively) [134]. Interestingly, all patients with a PR in both arms had MSS tumors, and no difference in PD-L1 expression by tumor proportion score (TPS) and combined positive score (CPS) was observed between responders and those who did not reach PR [134]. Similarly, a single-arm phase II trial demonstrated that patients with pretreated MSS mPDAC (*n* = 25) undergoing treatment with nivolumab/ipilimumab and fractionated radiotherapy achieved a DCR of 20% and an ORR of 12% [135]. No specific genomic pattern in tumor biopsies was predictive of response to treatment [135]. A comparable ORR of 5.1% was reported in a previously conducted phase I trial evaluating the efficacy of SBRT combined with durvalumab +/− tremelimumab in previously treated patients with mPDAC (*n* = 39) [136]. The phase II TRIPLE-R trial examined the efficacy of a combined radioimmunotherapy approach using ipilimumab, nivolumab, and the anti-interleukin-6 (IL-6) receptor Ab tocilizumab in combination with SBRT in patients with locally advanced or mPDAC and progressive disease after Gem or 5-FU-based therapy (*n* = 26) [137]. None of the patients enrolled in the trial had a clinical response. The mPFS and mOS were 1.6 and 4.9 months, respectively [137].

Using a mouse model of pancreatic cancer, Rech et al. demonstrated that radiation combined with ICI induced tumor shrinkage at the irradiated site but did not result in an abscopal effect at the unirradiated tumor site or prolonged survival [138]. However, when adding a CD40 agonist to ICIs and radiotherapy, not only a reduction in the irradiated tumor but also a decrease in the unirradiated tumor size was observed. Interestingly, despite T cell depletion, stable disease was still observed during treatment with ICIs, CD40 agonist and radiotherapy, suggesting that alternative mechanisms, such as activated myeloid cells, play a crucial role in maintaining tumor control [138]. In another pancreatic mouse model, Fujiwara et al. demonstrated that adding an IDO1 inhibitor to combined treatment with radiation and anti-PD-1 Ab did not result in enhanced antitumor efficacy compared to radiation therapy and anti-PD-1 Ab alone [139]. In conclusion, ICI in combination with radiotherapy can exert an increased anti-tumoral effect.

#### 3.7.2. Radiofrequency Ablation (RFA) Combined with ICI

RFA is an innovative procedure that induces local tumor necrosis by heat. Moreover, there is evidence from preclinical models that RFA induces an adaptive antitumor immune response through increased recruitment of T cells to the ablated zone [140]. Additionally, Shi et al. demonstrated that, in addition to the accumulation of TILs, RFA also enhanced PD-L1 expression in the TME of colorectal liver metastases [140]. However, experimental and clinical data on the role of RFA in PDAC are scarce. To the best of our knowledge, there are currently no studies that investigate a combined treatment approach consisting of RFA and ICI. In a small prospective study evaluating the effect of RFA on immune cells in patients with locally advanced pancreatic cancer (LAPC), peripheral blood was collected before and after the intervention [141]. After RFA, an increase in CD4^+^ and CD8^+^ T cells as well as effector memory T cells was observed between day 3 and day 30, whereas no difference in Tregs was observed. At day 30, the proportion of tumor antigen-presenting dendritic cells was enhanced. Additionally, “pro-tumorigenic” chemokines, such as CCL5, VEGF, TNFalpha, and others did not change after RFA [141]. Whether RFA in combination with ICI provides a meaningful clinical benefit in patients with mPDAC is unclear and needs to be explored.

#### 3.7.3. Irreversible Electroporation (IRE) Combined with ICI

An evolving technique for local tumor treatment is irreversible electroporation (IRE), a non-thermal, ablative procedure where tumors are exposed to pulses of electrical current. IRE induces tumor cell apoptosis and has been proven to be effective among patients with LAPC [142]. Despite the scarce evidence for IRE in mPDAC patients, preliminary data demonstrated that IRE combined with allogeneic NK cells prolonged survival [143,144]. The favorable clinical outcome of this combined treatment approach might be at least partly explained by its ability to stimulate a sustained and effective, systemic immune response. However, the exact mechanism for this local and systemic response is still unclear. A possible explanation might be that, contrary to thermal ablative procedures, IRE maintains the integrity of vessels within the ablated area, thereby facilitating the accumulation of TILs [145]. Cell debris with sustained neo-antigens resulted in increased antigen presentation to T cells through dendritic cells, enabling tumor control [146]. Furthermore, in comparison to radiofrequency ablation, IRE resulted in increased cytokine release, leading to enhanced local inflammation and more systemic effects [147]. Especially for mPDAC patients, systemic disease control that goes beyond the local IRE effect is of utmost importance.

Zhao et al. demonstrated that combined treatment with IRE and anti-PD-1 Ab resulted in increased infiltration of intratumoral CD8^+^ T cells and improved OS in a pancreatic cancer mouse model [148]. Interestingly, the addition of anti-CTLA-4 Ab to anti-PD-1 Ab and IRE did not result in longer survival compared to mice treated with IRE and anti-PD-1 [148].

Similarly, a recent study demonstrated that IRE triggered a systemic immune effect that inhibited secondary cancer growth in a syngeneic mouse model of PDAC. By adding ICIs to IRE, the systemic effects were even enhanced, resulting in a significant decrease in immunosuppressive MDSCs and a shift towards an increased ratio of immunostimulatory versus immunosuppressive M1 and M2 TAMs, respectively [149]. Additionally, a significant increase in CD8^+^ T cells and a decrease in Tregs were observed in the combined IRE and ICI treatment group vs. IRE-treated mice alone [149]. Narayanan et al. demonstrated that IRE combined with agonistic CD40 Ab prolonged survival in pancreatic cancer mouse models. Moreover, a combination of IRE with a toll-like receptor agonist (TLR7) and anti-PD-1 Ab inhibited the growth of concomitant distant tumors [150]. While depletion of CD8^+^ and CD4^+^ T cells completely abrogated the anti-tumoral effect of IRE, depletion of NK cells and macrophages merely attenuated the anti-tumoral activity of IRE [150]. These observations demonstrate that IRE is only effective in the presence of an intact immune system. IRE-treated tumors exhibited a significantly lower amount of MDSCs within the TME compared to untreated tumors in a murine pancreatic cancer model. Interestingly, no significant differences in CD4^+^ and CD8^+^ T cells or the TAM M1/M2 ratio were observed between IRE-treated and -untreated tumors. However, when IRE-pretreated mice were rechallenged with tumor cells on a contralateral site, no local tumor growth was observed [150]. This implies that IRE is capable of inducing protective immunity. When adding an anti-PD-1 Ab to IRE, no incremental benefit regarding tumor shrinkage or OS was achieved. However, the addition of a TLR7 agonist to combined anti-PD-1 and IRE treatment resulted not only in more pronounced local effects of IRE but also better systemic control [150]. In conclusion, experimental data indicate that IRE is capable of initiating a systemic immune response that can be further optimized by adding ICIs and TLR agonists in murine pancreatic cancer models. A phase II trial evaluated the safety and efficacy of the IRE of pancreatic liver metastases combined with nivolumab in patients with mPDAC. Due to low accrual, the study was prematurely terminated [151]. Currently, we still do not know whether adding an ICI to IRE might be sufficient to convert the immunosuppressive TME of pancreatic cancer into a “hot” immunoresponsive tumor. Therefore, a combined immunomodulatory approach, such as dual ICI together with targeted therapy and IRE might be promising to further advance in this field. An ongoing phase I trial is evaluating the safety of combined treatment with IRE and a TLR9 agonist with or without nivolumab in patients with pretreated mPDAC [152].

Table 2 provides an overview of early-phase clinical trials exploring ICIs with other immunomodulating strategies in mPDAC.

## 4. Future Perspective

Given the mainly disappointing results of single and dual ICI and ICI-based chemotherapy trials, new avenues have to be explored.

Since single and dual ICI have not translated into improved clinical outcomes in several trials, new ways have to be explored to optimize the treatment armamentarium against pancreatic cancer. Considering the stroma-rich TME with abundant immunosuppressive cells, such as Tregs, MDSCs, TAMs, and CAFs, a combined therapeutic approach comprising ICIs and agents simultaneously targeting immunosuppressive cells might be valuable to increase the clinical response and prolong survival in patients with PDAC.

First, we need to identify biomarkers that go beyond MSI-H, TMB-H, or high PD-L1 to better select the subgroups of mPDAC patients with a more “immunogenic” tumor profile who may derive benefit from an ICI-based therapy. Given the immunosuppressive TME of PDAC, ICIs have to be combined with further immuno-modulating agents targeting pro-tumorigenic components, such as CAFs, TAMs, MDSCs, and Tregs, to overcome the immune evasion of tumor cells. Additionally, therapeutic efforts should also focus on targeting the dense glycosaminglycane-rich extracellular matrix, which plays a twofold role in promoting tumor growth. It not only acts as a natural biological barrier to impair drug delivery to the tumor cells but also increases the immunosuppressive effect of the TME. Considering the diverse composition of the TME, including pro-tumorigenic and anti-tumorigenic components, we definitely need a more insightful understanding of the interaction complexity of cancer cells and the surrounding heterogenous TME, enabling us to better identify druggable targets within the tumor stroma and the TIME. Combined therapeutic approaches consisting of ICIs, targeted agents, oncolytic viruses or genetically modified attenuated bacteria, vaccinations, cellular therapies, and backbone chemotherapy are crucial to enlarging our treatment armamentarium against pancreatic cancer.

## Figures and Tables

**Table 1:** A selection of completed ICI-based phase II trials in mPDAC.

Trial	Phase	Treatment	Setting	N	Primary and Secondary Outcomes	Results	Ref.
CCTG PA.7 NCT02879318	II	Gemcitabine + nab-paclitaxel + durvalumab + tremelimumab vs. Gemcitabine + nab-paclitaxel	First-line	180	1. mOS 2. mPFS, ORR	mOS: 9.8 vs. 8.8 months (HR: 0.94, 90% CI, 0.71–1.25), *p* = 0.72 mPFS: 5.5 vs. 5.4 months (HR: 0.98, 90% CI, 0.75–1.29), *p* = 0.91 ORR: 30.3% vs. 23.0%, *p* = 0.28	[41]
CISPD3	II	Sintilimab + mFOLFIRINOX vs. mFOLFIRINOX	First- or second-line	110	1. mOS 2. mPFS, ORR	mOS: 10.9 vs. 10.8 months (HR: 1.07, 95% CI, 0.69–1.68), *p* > 0.05 mPFS: 5.9 vs. 5.7 months (HR: 0.93, 95% CI, 0.62–1.40), *p* > 0.05 ORR: 50% vs. 23.9%, *p* < 0.05	[42]
PRINCE	II	Arm 1: Gemcitabine + nab-paclitaxel + nivolumab Arm 2: Gemcitabine + nab-paclitaxel + sotigalimab Arm 3: Gemcitabine + nab-paclitaxel + nivolumab + sotigalimab	First-line	105	1. 1-year OS vs. historical control rate of 35% 2. mPFS, ORR, DCR, DOR	Arm 1: 1-year OS: 57.7%, *p* = 0.006 mOS: 16.7 months mPFS: 6.4 months Arm 2: 1-year OS: 48.1%, *p* = 0.062 Arm 3: 1-year OS: 41.3%, *p* = 0.223	[43]
JapicCTI-184230	II	mFOLFIRINOX + nivolumab	First-line	31	1. ORR 2. mOS, mPFS	ORR: 32.3% (CR: 0.%, PR: 32.3%) mOS: 13.4 months mPFS: 7.4 months	[44]
NCT01896869	II	FOLFIRINOX followed by ipilimumab + GVAX vs. FOLFIRINOX	Maintenance after 8–12 cycles first-line FOLFIRINOX	82	1. mOS 2. mPFS	mOS: 9.4 vs. 14.7 months (HR: 1.75, 95% CI, 1.09–2.79), *p* = 0.019 mPFS: 2.4 vs. 5.6 months (HR: 2.92, 95% CI, 1.70–5.02), *p* < 0.001	[45]
NCT02558894	II	4 cycles durvalumab + tremelimumab, followed by durvalumab vs. durvalumab monotherapy, up to 12 months	Second-line	65	1. ORR 2. mPFS, mOS	ORR: 3.1% vs. 0% mPFS: 1.5 vs. 1.5 months mOS: 3.1 vs. 3.6 months	[46]
NCT02077881	II	Gemcitabine + nab-paclitaxel + indoximod	First- or second-line	104	1. mOS 2. ORR	mOS: 10.9 months ORR: 46.2%	[47]
NCT02331251	Ib/II	Gemcitabine + nab-paclitaxel + pembrolizumab	First-or second line	17	1. >15% CR 2. mOS, mPFS	ORR: 17.6% (0 CR + 3 PR) mOS: 15.0 months mPFS: 9.1 months	[48]
NCT00112580	II	Ipilimumab monotherapy	First-/second-/or further-line	27	ORR	ORR: 0%	[49]

mFOLFIRINOX, modified FOLFIRINOX; mOS, median overall survival; mPFS, median progression-free survival; ORR, objective response rate; HR, hazard ratio; CI, confidence interval; DCR, disease control rate; DOR, duration of response; GVAX, granulocyte–macrophage colony-stimulating factor-allogeneic pancreatic tumor cells; CR, complete remission; PR, partial remission.

**Table 2:** A selection of early phase clinical trials investigating ICI in combination with other immunomodulatory strategies in mPDAC.

Combination Strategy	Phase	Setting	N	Drugs/Intervention	Efficacy and Survival Data	Potential Mechanism
ICI + PARP inhibitor [65]	I/II	Maintenance after 4 months of platin-based treatment in LAPC and mPDAC	84	Anti-PD1 Ab nivolumab + niraparib Anti-CTLA4 Ab ipilimumab + niraparib	mPFS at 6 months: 20.6% vs. 59.6% mOS: 13.2 vs. 17.3 months ORR: 7.7% vs. 15.4%	Increased intratumoral CD8^+^ activity following treatment with anti-CTLA4 Ab and PARP inhibitor
ICI + anti-CSF1R inhibitor [71]	I/II	Second or further-line	27	Anti-PD-1 Ab pembrolizumab + CSF1R inhibitor	ORR: 3.7% mPFS: 1.4 months mOS: 2.2 months	Inhibition of TAMs and MDSCs
ICI + anti-CCR4 Ab [88]	I	Second or further-line	24	Anti-PD-L1 Ab durvalumab + anti-CCR4 Ab mogamulizumab vs. Anti-CTLA4 Ab tremelimumab + anti-CCR4 Ab mogamulizumab	ORR: 0% vs. 0%	Targeting CCR4 in combination with ICI results in a decrease in Tregs and an increased amount of CD8^+^ T cells
ICI + CXCR4 antagonist [94]	II	Second or further- line	29	CXCR4 antagonist motixafortide + anti-PD-1 Ab pembrolizumab	ORR: 3.4% DCR: 34.5% mOS: 3.3 months	Increase in intratumoral CD8^+^ T cells and a reduction in MDSCs
ICI + TGFbeta inhibitor [107]	I	Second or further-line	32	Anti-PD-L1 Ab durvalumab + TGFbeta receptor I kinase inhibitor galunisertib	mPFS: 1.9 months mOS: 5.7 months ORR: 3.1%	ICI and anti-TGFbeta enhance effector T cell activity
ICI + Bruton tyrosine kinase inhibitor [115]	II	Second or further-line	73	Acalabrutinib vs. Anti-PD-1 Ab pembrolizumab + acalabrutinib	ORR: 0% vs. 7.9% DCR: 14.3% vs. 21.1% mPFS: 1.4 vs. 1.4 months mOS: 3.6 vs. 3.8 months	Combined treatment decreases MDSCs and activates CD4^+^ and CD8^+^ T cells
ICI + MET kinase inhibitor [123]	I	Further-line	17	PD-L1 inhibitor + MET kinase inhibitor merestinib	ORR: 20% (dose-escalation cohort) 0% (expansion cohort)	ICI + MET kinase inhibitor enhances T cell response and decreases immunosuppressive effects of MDSCs
ICI + oncolytic viruses [126]	I	Further-line	11	Anti-PD-1 Ab pembrolizumab + pelareorep + chemotherapy	ORR: 9% mPFS: 2.0 months mOS: 3.1 months	Enhances T cell migration to the TME
Radiotherapy + ICI [134]	II	Further-line	84	SBRT + nivolumab vs. SBRT + nivolumab + ipilimumab	DCR: 17.1% vs. 37.2% mPFS: 1.7 vs. 1.6 months mOS: 3.8 vs. 3.8 months	Radiotherapy + ICI induce an antitumoral effect by increasing CD8^+^ T cells and CD8^+^/Treg ratio while decreasing MDSCs

Abbreviations: mPDAC = metastatic pancreatic cancer, ICI = immune checkpoint inhibitor, LAPC = locally advanced pancreatic cancer, mPFS = median progression-free survival, mOS = median overall survival, ORR = objective response rate, DCR = disease control rate, TAMs = tumor-associated macrophages, MDSCs = myeloid-derived suppressor cells, TME = tumor microenvironment, Tregs = regulatory T cells.
